# “One-stop” Hybrid approach for tetralogy of fallot with aortopulmonary collateral arteries in adults

**DOI:** 10.12669/pjms.292.3192

**Published:** 2013-04

**Authors:** Yiyao Jiang, Wei Zhang, Yu Pei, Li Yang, Langang Xue, Xiaocheng Liu

**Affiliations:** 1 Yiyao Jiang, Tianjin Medical University, 3,4,6: Peking Union Medical College, Chinese Academy of Medical Sciences, Department of Cardiovascular Surgery,TEDA International Cardiovascular Hospital, 61# Third Avenue Tianjin Economic Development Area, Tianjin, 300457, China.; 2 Wei Zhang, Tianjin Medical University, 3,4,6: Peking Union Medical College, Chinese Academy of Medical Sciences, Department of Cardiovascular Surgery,TEDA International Cardiovascular Hospital, 61# Third Avenue Tianjin Economic Development Area, Tianjin, 300457, China.; 3 Yu Pei, Department of Cardiovascular Surgery,TEDA International Cardiovascular Hospital, 61# Third Avenue Tianjin Economic Development Area, Tianjin, 300457, China.; 4 Li Yang, Tianjin Medical University, 3,4,6: Peking Union Medical College, Chinese Academy of Medical Sciences, Department of Cardiovascular Surgery,TEDA International Cardiovascular Hospital, 61# Third Avenue Tianjin Economic Development Area, Tianjin, 300457, China.; 5 Langang Xue, Department of Cardiovascular Surgery,TEDA International Cardiovascular Hospital, 61# Third Avenue Tianjin Economic Development Area, Tianjin, 300457, China.; 6 Xiaocheng Liu, Tianjin Medical University, 3,4,6: Peking Union Medical College, Chinese Academy of Medical Sciences, Department of Cardiovascular Surgery,TEDA International Cardiovascular Hospital, 61# Third Avenue Tianjin Economic Development Area, Tianjin, 300457, China.

**Keywords:** Tetralogy of fallot, Aortopulmonary collateral arteries, “One-stop” hybrid

## Abstract

We report a 45 year-old Chinese woman with tetralogy of fallot that had two aortopulmonary collateral arteries and tricuspid regurgitation. Collateral circulation was blocked and total correction was successfully performed in our “one-stop” hybrid operation room. The patient was weaned from cardiopulmonary bypass after 97 minutes and was transferred to the intensive care unit for about 36 hours. Without any complications, the patient was discharged home in the following eight days.

## INTRODUCTION

Tetralogy of fallot with aortopulmonary collateral arteries (TOF-APCAs) is a common complex congenital heart disease. Due to the existence of aortopulmonary collateral arteries, it is difficult to operate and prone to the occurrence of complications, such as respiratory distress syndrome, low cardiac output syndrome and heart failure. Recently, because of its procedural simplicity, safety and lower cost, “one-stop” Hybrid procedures have been applied to the treatment of cardiovascular diseases gradually. Here, we describe one case with the application of “one-stop” hybrid operations for TOF-APCAs in adults.

## CASE REPORT

A 45-year-old (45kg) female patient was admitted to our department complaining of a 40-year history of the cyanotic lips and lower extremity edema occasionally. On physical examination, a systolic 3/6 rough murmur was heard at left sternal edge, clubbed nails of fingers (toes) were found. Transthoracic echocardiography showed moderately dilated right atrium and ventricle with septal defect measuring approximately 2.2 cm x 2.0 cm, aortic riding 50%, pulmonary artery stenosis with two leaflets, dilation of the tricuspid valve annulus and tricuspid regurgitation.

**Fig.1:**
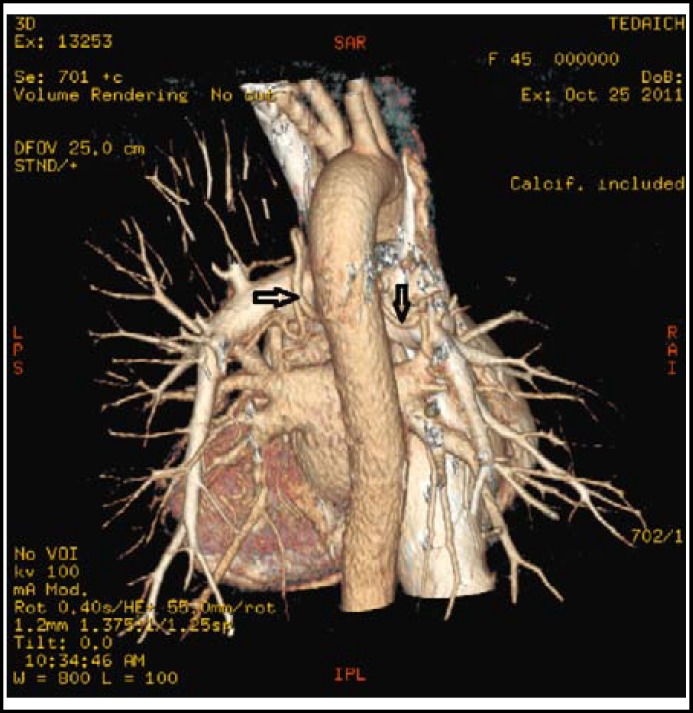
The formation of aortopulmonary collateral arteries (black arrow

The chest computed tomography scan revealed the formation of aortopulmonary collateral arteries and the ratio of McGoon approximately equaled to 1.66 (Fig.1). According to the results of examinations, the diagnosis was congenital heart disease, tetralogy of fallot with aortopulmonary collateral arteries and tricuspid regurgitation. After discussion, we decided to apply “one-stop” hybrid approach for the patient in our hybrid operation room, block the aortopulmonary collateral arteries with interventional transcatheter and then perform tetralogy of fallot radical mastectomy.

**Fig.2:**
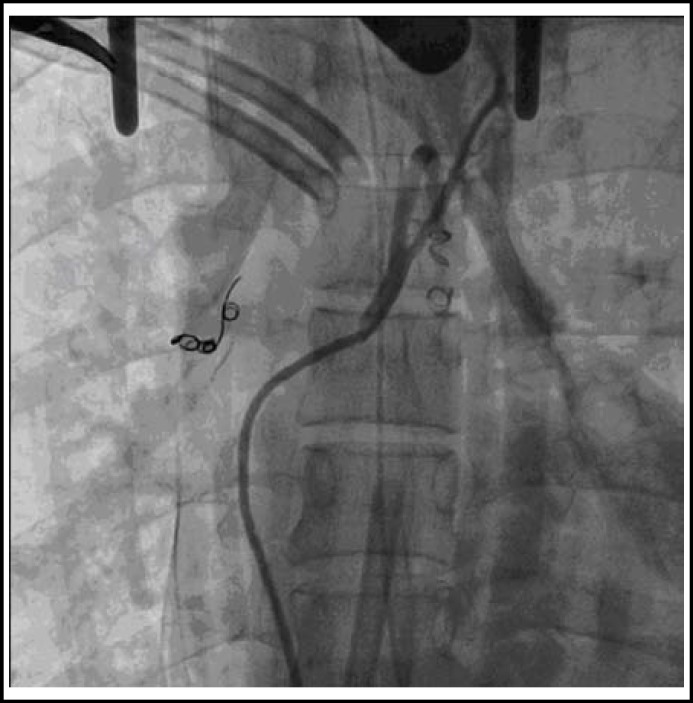
No residual shunt was found after two spring embolism placed

Under general anesthesia with a double lumen endotracheal tube, median sternotomy thoracic, aorta and right atrium pockets were performed. We placed a 6F Judkins Right 4.0 angiography catheter to carry out the angiography of aortopulmonary collateral arteries through 6F sheath via the right femoral artery. Two collateral arteries were derived from the descending aorta anterior wall to the left and right pulmonary arteries, respectively. After placing spring emboli to the left one, the angiography verified that no residual shunt existed. Optitorque catheter was placed at the proximal of right collateral artery via guiding catheter 6F Judkins Right 1.5, then set the microcatheter progreat to the middle of collateral artery, after two spring embolism placed, there was no residual shunt (Fig.2). When the physician intervention was completed, the cardiopulmonary bypass was established by surgeon routinely.

Longitudinal incision was made at right ventricular outflow tract, the thickening of the septum and the wall-beam were cut off, then the junction of the pulmonary valve was cut, the right ventricular outflow tract was clear. Widen the pulmonary artery with autologous pericardium. Fossa ovalis was closed with single prolene stitch. Tricuspid valvoplasty was performed with 2/0 Ethibond. Continuous suture closed the incision of right atrium with 5-0 prolene. Declamping the ascending aorta and vena cava, the patient was weaned from cardiopulmonary bypass after 97 minutes and was transferred to the intensive care unit for about 36 hours.

Echocardiography was undertaken on postoperative day 6 and the patient was discharged home the following day without any complications.

## DISSCUSION

Tetralogy of fallot with aortopulmonary collateral arteries in adults is complex in the pathophysiological condition, varies in the anatomical location.^1^ If the surgical ligation APCAs is applied, it might result in a greater surgical trauma and need superb surgical skills. Even some patients can not receive surgical treatment due to large APCAs. Therefore, the problem that how to deal with the APCAs effectively has become one of the key factors to the TOF-APCAs surgical success and prognosis.

Hybrid technology is applied into APCAs for a long time. However, it is used as a staging treatment strategies or postoperative intervention.^2^ It includes: blocking the APCAs in the catheter lab, then deliver the patient to operation room immediately or wait to surgery for some days; during the operation, look for APCAs and ligate them, then carry on surgical treatment. If the signs of pulmonary exudative lesions appear after operation, it is necessary to undergo angiography and occlude the APCAs. While there are some constraints to staging hybrid, for example, repeated anesthesia and transfer patient between the catheter lab and operative room. It might induce preoperative risk increased, long hospital stay and high medical cost. For postoperative intervention of APCAs, many complications may occur because of pulmonary exudative lesions or prolonged mechanical ventilation time.

In recent years, “one-stop” hybrid operation is applied into cardiovascular surgery gradually, one of its feature is that the diagnostic imaging, interventional treatment and heart surgery are completed in the same operating table at the same time.^3^ It will avoid the potential risks brought by repeated anesthesia and reduce the repeated hospitalization and prolonged treatment.^4^

For TOF-APCAs, “one stop” hybrid can make intervention and surgery achieve "zero gap" convergence. This hybrid combination guarantees the prevention of the damaging effects of hypoxemia after APCA occlusion, while the patients is being shifted to operation room. In addition, the patient who has “one-stop” hybrid operation will recover well because of the shorter ICU stay and intubation time, and the patient’s hospitalization time and costs are lower.^5^ It indicates that “one-stop” hybrid technology can promote TOF-APCAs patients postoperative recovery effectively and reduce the economic burden of patients.

In conclusion, "one-stop" hybrid technology allows interventional treatment and surgical treatment integration. It simplifies the treatment process, promotes patients’ postoperative recovery and reduces the psychological and economic burden of angiography and surgery. Although “one-stop” hybrid technology will benefit more patients, it should be noted that this technology is still in the initial stage of exploration and need to accumulate more experience in order to achieve better outcomes.

## Authors contribution

Yiyao Jiang, Wei Zhang and Langang Xue performed surgery, Yiyao Jiang wrote this case report. As the corresponding author, Xiaocheng Liu revised the section of discussion in this article and directed Yiyao Jiang to complete the case report. Yu Pei and Li Yang collected the data and pictures in CT and angiography.
